# Seed germination prediction of *Salvia limbata* under ecological stresses in protected areas: an artificial intelligence modeling approach

**DOI:** 10.1186/s12898-020-00316-4

**Published:** 2020-08-29

**Authors:** Maryam Saffariha, Ali Jahani, Daniel Potter

**Affiliations:** 1 Rangeland Management, College of Natural Resources, University of Tehran, Tehran, Iran; 2 Faculty of Natural Environment and Biodiversity Department, College of Environment, Standard Square, Karaj, Iran; 3 Department of Plant Sciences, College of Agricultural and Environmental Sciences, University of California Davis, Davis, USA

**Keywords:** Ecological stress, Multi-layer perceptron, Multiple linear regression, Neural network, Seed germination, *Salvia limbata*

## Abstract

**Background:**

*Salvia* is a large, diverse, and polymorphous genus of the family Lamiaceae, comprising about 900 ornamentals, medicinal species with almost cosmopolitan distribution in the world. The success of *Salvia limbata* seed germination depends on a numerous ecological factors and stresses. We aimed to analyze *Salvia limbata* seed germination under four ecological stresses of salinity, drought, temperature and pH, with application of artificial intelligence modeling techniques such as MLR (Multiple Linear Regression), and MLP (Multi-Layer Perceptron). The *S.limbata* seeds germination was tested in different combinations of abiotic conditions. Five different temperatures of 10, 15, 20, 25 and 30 °C, seven drought treatments of 0, −2, −4, −6, −8, −10 and −12 bars, eight treatments of salinity containing 0, 50, 100.150, 200, 250, 300 and 350 mM of NaCl, and six pH treatments of 4, 5, 6, 7, 8 and 9 were tested. Indeed 228 combinations were tested to determine the percentage of germination for model development.

**Results:**

Comparing to the MLR, the MLP model represents the significant value of R^2^ in training (0.95), validation (0.92) and test data sets (0.93). According to the results of sensitivity analysis, the values of drought, salinity, pH and temperature are respectively known as the most significant variables influencing *S. limbata* seed germination. Areas with high moisture content and low salinity in the soil have a high potential to seed germination of *S. limbata*. Also, the temperature of 18.3 °C and pH of 7.7 are proposed for achieving the maximum number of germinated *S. limbata* seeds.

**Conclusions:**

Multilayer perceptron model helps managers to determine the success of *S.limbata* seed planting in agricultural or natural ecosystems. The designed graphical user interface is an environmental decision support system tool for agriculture or rangeland managers to predict the success of *S.limbata* seed germination (percentage) in different ecological constraints of lands.

## Background

*Salvia* is a large, diverse, and polymorphous genus of the family Lamiaceae, comprising about 900 ornamentals, medicinal species with almost cosmopolitan distribution in the world [1–3]. In fact, 58 species of this genus are found in Iran as 17 species are endemic [4, 5]. Some species of the genus, e.g., *Salvia officinalis*, possess economic importance as flavoring agents in perfumery, seasoning and cosmetology. *S. limbata* has been credited with a long list of medicinal uses: spasmolytic, antiseptic, astringent, antibiotic, creaminess, liver stimulant and digestive enhancer as some of the phenolic compounds of plants belonging to this genus, have also shown excellent antimicrobial activity [6–9].

In Iran, *Salvia limbata* is highly endangered under over grazing and exploitation by local people and the climate changes phenomena. *Salvia limbata* is widely used in traditional medicine for its therapeutic effects. The success of seed germination is one of the main economic and ecological solutions to preserve the medicinal plants such as *Salvia limbata*. However, the success of seed germination depends on a numerous ecological factors and stresses such as salinity stress [10, 11], drought stress [12, 13], temperature [14, 15], and pH [11, 12]. Salinity, as an abiotic ecological factor, limits crops production and the chance of seed germination [16]. Water absorbency will be diminished under salinity stress in which several metabolic and physiological processes are influenced and the duration of seed germination is prolonged [17]. Temperature, with affecting the enzymatic activity, highly influences seed germination and determine the periodicity of seed germination [18, 19]. Researchers [18, 20, 21] believe that germination rate increases when temperature meets an optimal level, and then it declines sharply. Other factors such as drought and pH also affect seed germination in many species [22, 23].

Quantitative knowledge of the salinity stress, drought stress, temperature and pH that influences *Salvia limbata* seed germination is scarce. Most of the researches have been focused on regression models in prediction of seed germination under temperature [12, 20, 24, 25] and drought stresses [26, 27]. In other researches thermal time models are common methods to determine the seed germination rate under ecological stresses [28]. In thermal time models, seeds should accumulate thermal-time unit which is commonly degree-days, for a percentage of the population to germinate. In below optimum temperature, seeds germination rate starts to decrease and above optimum temperature seeds germination rate increases linearly with temperature [28]. Recent researches commonly developed hydrothermal models in seed germination of agricultural or wild species using linear or non-linear regression models [29–32]. These models determine base, optimum and maximum ecological conditions for seed germination. For instance, Duarte et al. [28] determined the thermal time requirement for seed germination for bromeliads during different climatic periods. Base time from 6.2 to 10 °C and ceiling time from 31.6 to 41.7 °C were identified, depending on the species. In other thermal time study, Galindez et al. [33] applied thermal time model combined with darkness to assess germination base temperature in photoblastic seeds of three aromatic–medicinal Verbenaceae species in genera Lippia and Aloysia. The results revealed that some species seed germination only occur in light conditions, while seed germination in some other species, was significantly more successful in the light than in darkness. Indeed, in these models, researchers treated seeds with at most two ecological factors such as water and temperature (hydrothermal model) or light and temperature (light-thermal model). New artificial neural network models have potential to test multiple ecological conditions or stresses on seed germination simultaneously to determine the results of different combinations of ecological stresses on seed germination rate.

Recently, artificial intelligence techniques such as Multiple Linear Regression (MLR) and Multi-Layer Perceptron (MLP) neural network, as nonlinear and dynamic modeling techniques, have been employed for the development of accurate models in environmental sciences [34–36]. The new quantitative modeling methods and artificial intelligence approach are essential to deal with ecological phenomena [37, 38] such as seed germination. MLP modeling technique has been developed based on the structure of the human brain in data processing. A variety of mathematical functions have been applied in artificial intelligence modeling techniques to increase the ability of model in output prediction. For example, Jahani [35] proved that artificial neural network with multi-layer perceptron predicts the quality of forest landscapes with high accuracy in comparison with multiple regression models. In this research we aimed to analyze *Salvia limbata* seed germination under four ecological stresses of salinity, drought, temperature and pH, with application of artificial intelligence modeling techniques such as MLR, and MLP. The sensitivity of the most accurate model will prioritize ecological factors in *Salvia limbata* seed germination. Finally, the Graphical User Interface (GUI) tool as an environmental decision support system will be provided for ecologists to predict the percentage of seed germination of *Salvia limbata* in the field.

## Results

In this research, two predictive models which are multiple regression and ANN model were investigated to compare results in *S. limbata* seed germination.

### Multiple linear regression model (MLR)

To avoid limitation in model generalization, 80% of total samples (228 treatments) were selected randomly for multiple regression modeling and the resulted statistical indices have been presented in Table 1. The remained samples (46 treatments = 20%) were applied to test the result of regression equation (Eq. 8) and detecting the generalization potential of regression equation. Regression equation was applied to predict the seed germination in test data.

**Table 1:** Statistical indices of MLR model in training and test sets

Model	Performance measures	Training set	Test set	All data
MLR	R^2^	0.66	0.73	0.67
	MSE	199.2	125.8	140.6
	RMSE	14.11	11.22	11.86
	MAE	11.94	9.27	9.81

8 $$\varvec{PSG} = 3.139 \times D - 0.077 \times S + 0.065 \times {\text{T}} + 0.325 \times {\text{P}} + 42.739$$

In Eq. 8, PSG is the percentage of seed germination, D is drought, S is salinity, T is temperature and P is pH.

**Fig. 1:**
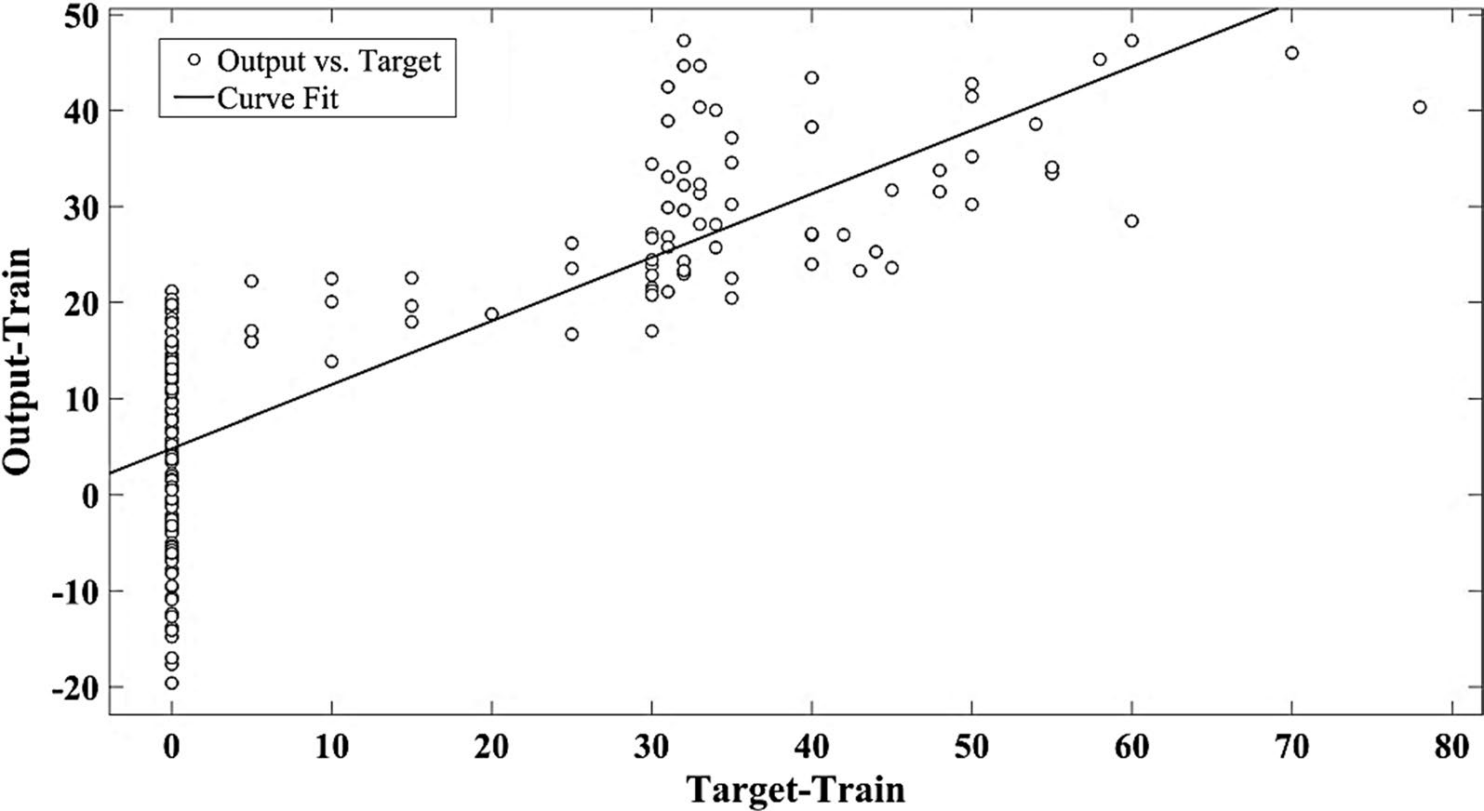
The scatter plot of target versus predicted seed germination by MLR in train data set

**Fig. 2:**
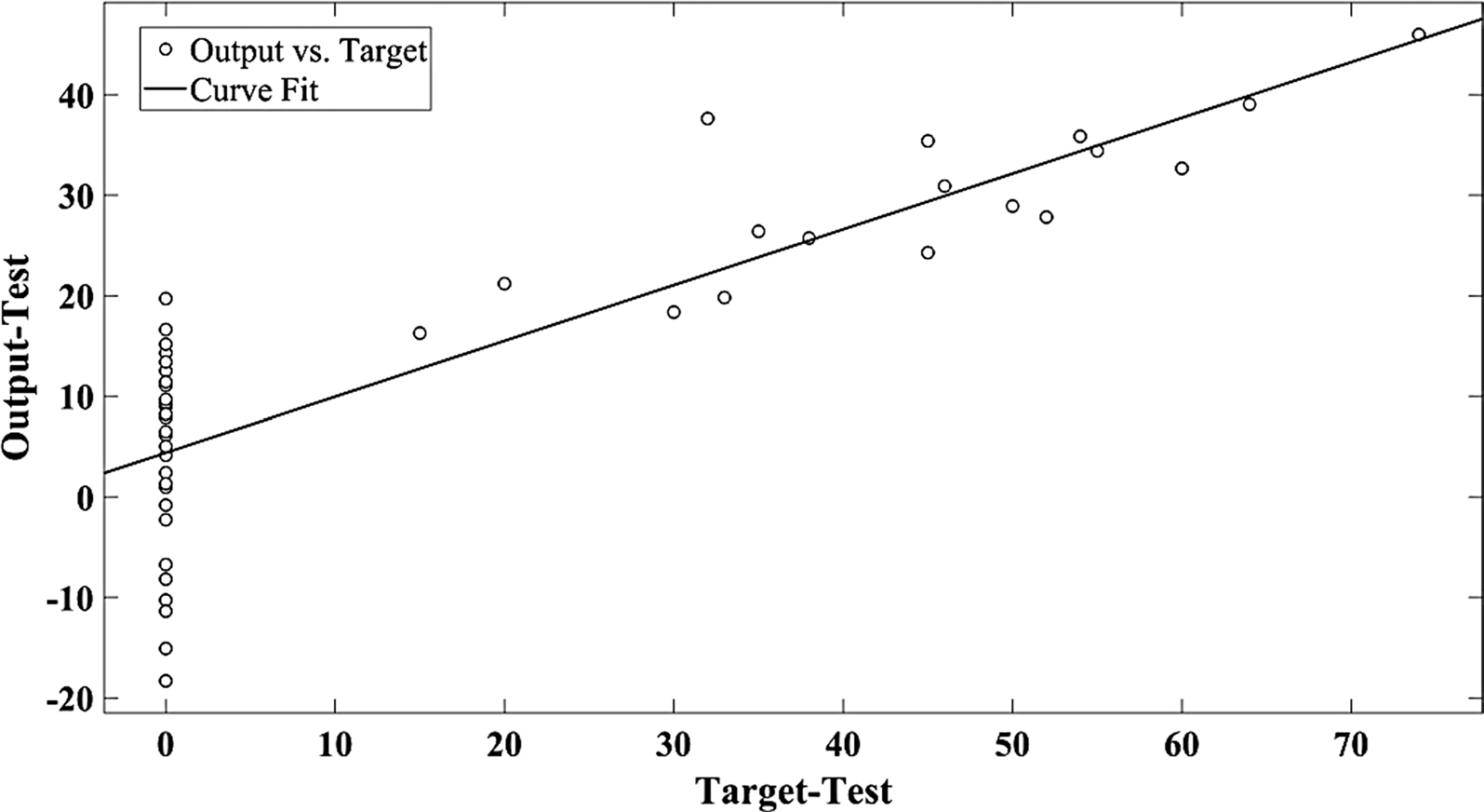
The scatter plot of target versus predicted seed germination by MLR in test data set

**Fig. 3:**
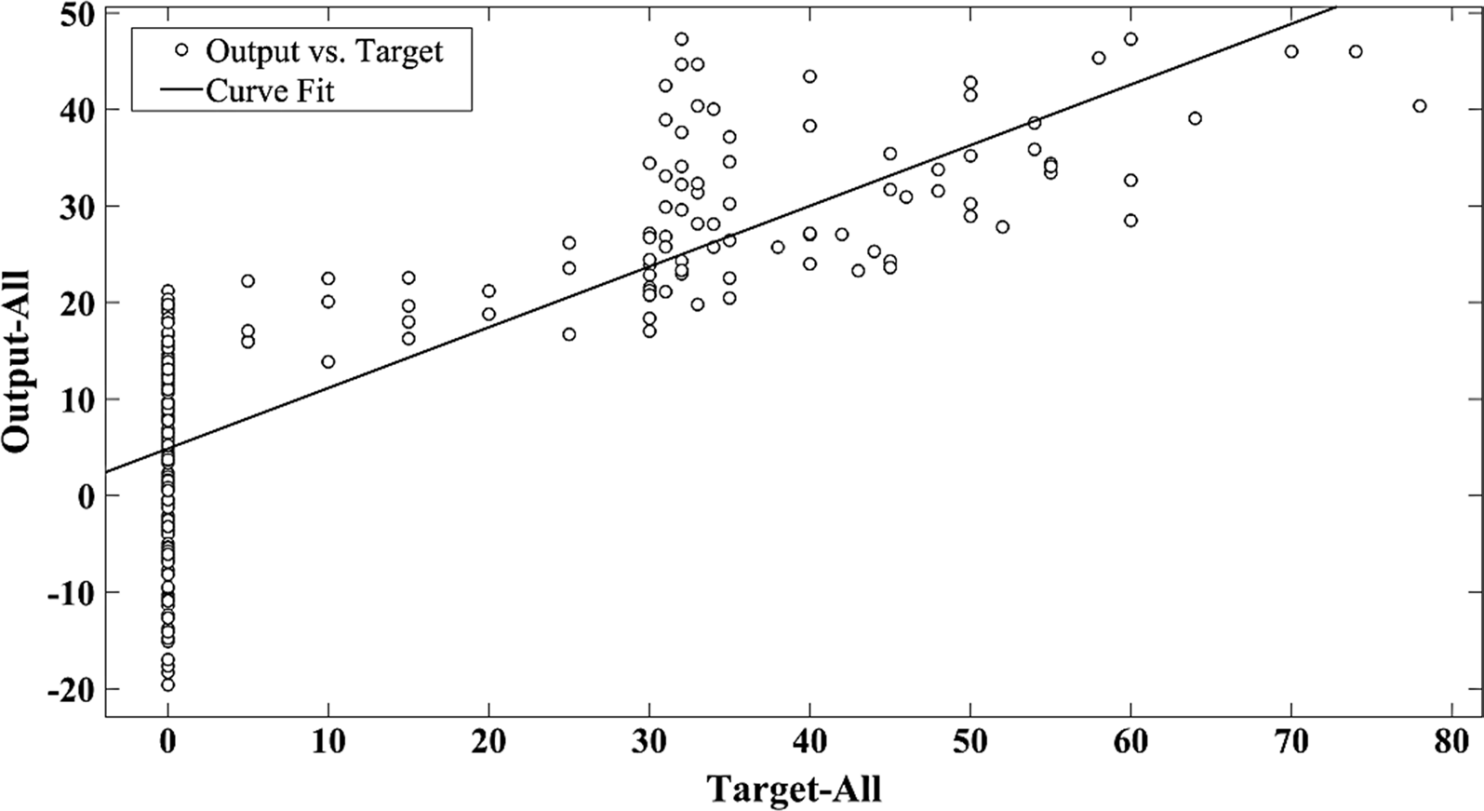
The scatter plot of target versus predicted seed germination by MLR in All data set

The results of MLR model’s accuracy in training and test samples have been summarized in Table 1 by R^2^, MSE, RMSE and MAE indices. The correlation of MLR outputs and target seed germination has been illustrated in Figs. 1, 2 and 3 using linear regression model.

### MLP modeling

The abiotic influential variables, as input variables, and *S.limbata* seed germination as outputs, were summarized in the MATLAB R2018b software to design the most accurate structure of MLP. The 228 treatments were used to train the feed forward neural networks. The recorded data contain 4 influential variables as input data sets in designed MLP and seed germination as output data sets.

The structure of optimized MLP was obtained using determination of the activation function, the number of hidden layers and its’ neurons and the most accurate predictive model has been achieved by the MLP technique (Table 2).

**Table 2:** The performance measures of the best MLPs in test phase

Activation function	Training function	Structure	Accuracy	Train	Validation	Test	All
*Logsig-logsig-purelin*	*LM*	*4-10-10-1*	R^2^	*0.95*	*0.92*	*0.93*	*0.94*
			MSE	*16.26*	*36.53*	*30.86*	*25.22*
			RMSE	*4.03*	*6.04*	*5.55*	*5.02*
			MAE	*2.57*	*3.85*	*3.33*	*3.18*
Logsig- purelin	GDM	4-7-1	R^2^	0.92	0.90	0.90	0.91
			MSE	35.55	54.65	55.12	43.44
			RMSE	5.96	7.39	7.42	5.87
			MAE	3.65	4.11	4.14	3.95
Tansig- Tansig- purelin	BFG	4-12-12-1	R^2^	0.90	0.91	0.91	0.91
			MSE	54.15	43.24	44.32	42.14
			RMSE	7.36	6.58	6.66	6.49
			MAE	4.09	3.94	3.98	3.92
Logsig- purelin	CGB	4-10-1	R^2^	0.94	0.90	0.91	0.92
			MSE	23.34	53.33	44.54	34.45
			RMSE	4.83	7.3	6.67	5.87
			MAE	3.05	4.03	4.01	3.51
Tansig-Tansig-purelin	GDA	4-9-9-1	R^2^	0.92	0.94	0.93	0.93
			MSE	36.56	25.35	31.44	32.12
			RMSE	6.05	5.03	5.61	5.67
			MAE	3.91	3.05	3.43	3.47
Tansig-Tansig-purelin	CGP	4-13-13-1	R^2^	0.92	0.93	0.93	0.93
			MSE	35.15	30.67	30.45	31.23
			RMSE	5.93	5.54	5.52	5.59
			MAE	3.61	3.31	3.29	3.4

Italics values: Optimum MLP for prediction of seed germination

According to the values of R^2^ (Table 2), the structure of “4-10-10-1″ was defined as the optimized MLP model and the most successful topology in the seed germination prediction of *S.limbata*. The defined topology includes four ecological parameters as inputs, 10 neurons in the structure of hidden layers and 1 neuron to calculate the percentage of seed germination in the output layer. As we found, the logarithm sigmoid is the most successful function in hidden layer and linear transfer function is the most proper function in output layer to achieve the most accurate seed germination prediction.

**Fig. 4:**
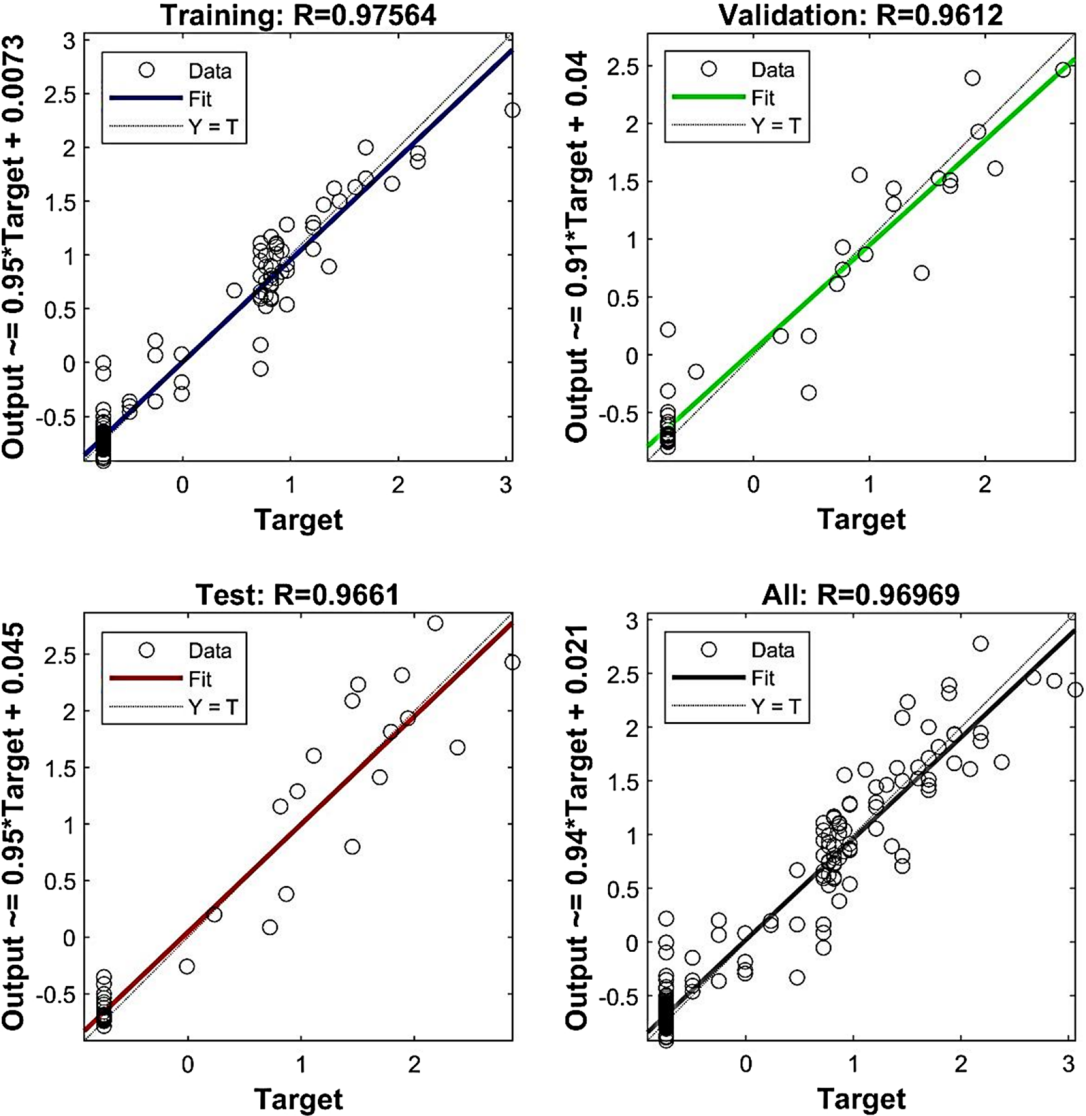
Scatter plots of output versus target seed germination values by MLP

The scatter plot will be applicable to illustrate correlation between variables [41, 42, 55]. The scatter plot in Fig. 4 illustrates the differences between outputs of MLP model and target seed germination in four data sets. The value of coefficient determination reveals a significant correlation between target seed germination and MLP outputs.

**Fig. 5:**
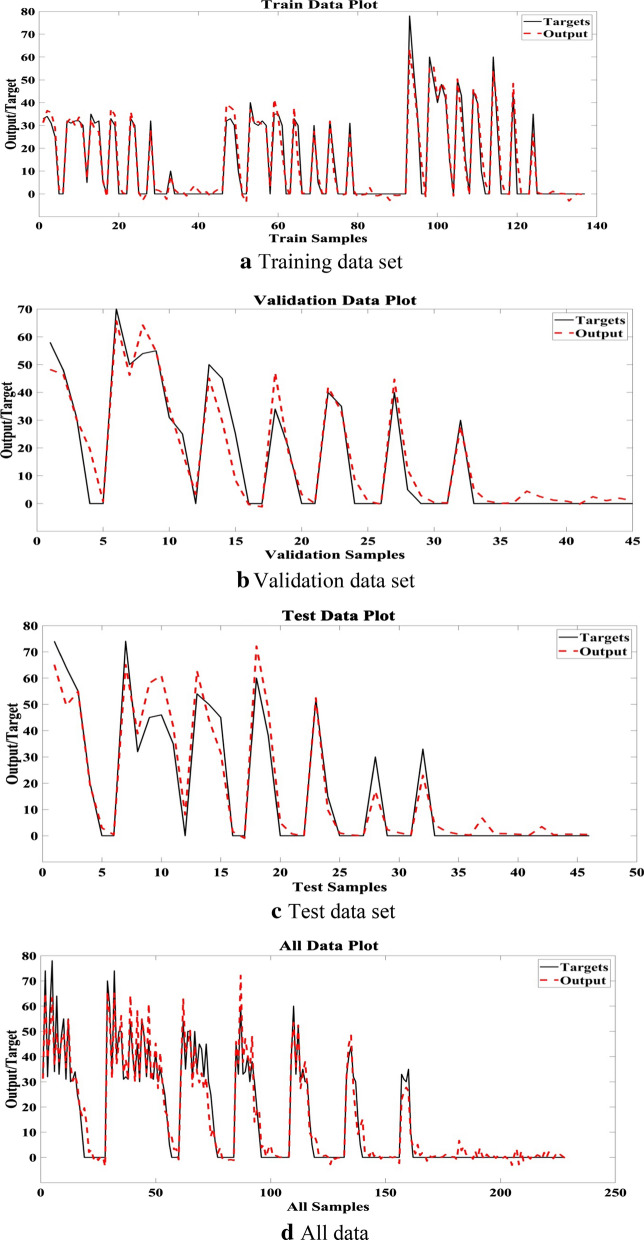
Target and simulated seed germination values by MLP

Figure 5 compares the real and simulated values of seed germination percentage in the training, validation, test and all data. A meaningful and distinctive agreement between target and simulated values is illustrated in Fig. 5.

Considering results of different designed structure of ANNs, MLP model with significant value of R^2^, makes more accurate correlation between inputs and outputs. So, S.*limbata* seed germination model, using of 4 abiotic variables, as model input variables, can be more accurate model for S.*limbata* seed germination prediction. The Eq. 9 is proposed as S.*limbata* seed germination model.

9 $${\text{MLP}} = purelin\left\{ {logsig\left\{ {\sum LW_{2,1} \left\{ {logsig\left( {\sum IW_{1,1pi} + b_{1} } \right)} \right\} + b_{2} } \right\}} \right\}.$$

where, MLP: Multi-layer perceptron model, p_i_: inputs signals, LW_ji_: layer weights, IW_ji_: indicates the input weights, ∑b_i_: represents the biases, i is the neuron number and j is layer number.

As the results reveal, MLP model (R^2^ = 0.94) is more accurate than MLR (R^2^ = 0.67). Therefore we focus on the analysis of MLP model for model variables prioritization.

### Sensitivity analysis of MLP

**Fig. 6:**
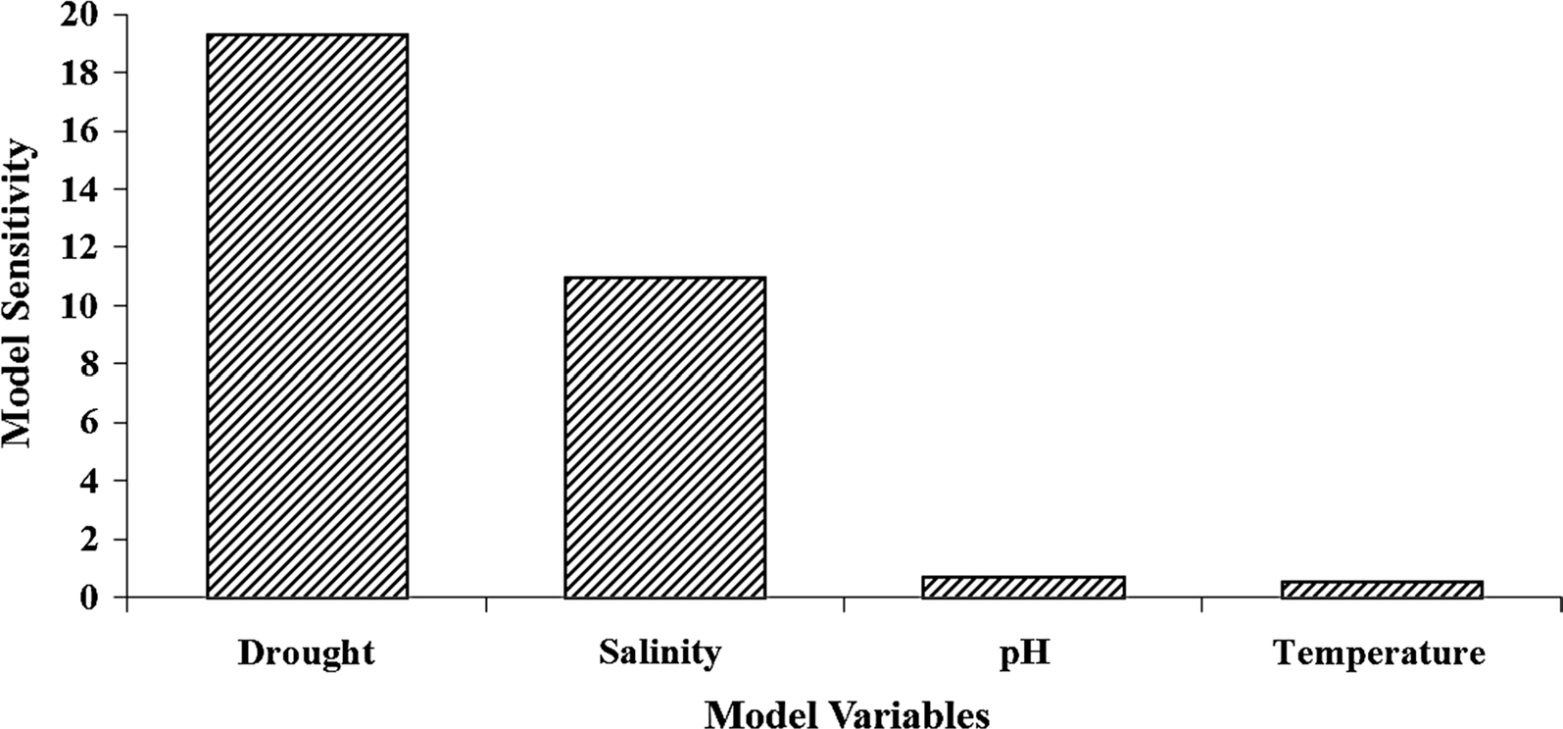
The results of sensitivity analysis of MLP

For sensitivity analysis of the most accurate model, which is MLP model, each input variable was withdrawn while not manipulating any of the other variables and then the MLP model was trained for every pattern. According to results in Fig. 6, the share of each input variable of developed MLP on desired output can be realized clearly. According to sensitivity analysis, the values of drought, salinity, pH and temperature are known as the most significant variables which influence *S. limbata* seed germination (Fig. 6).

**Fig. 7:**
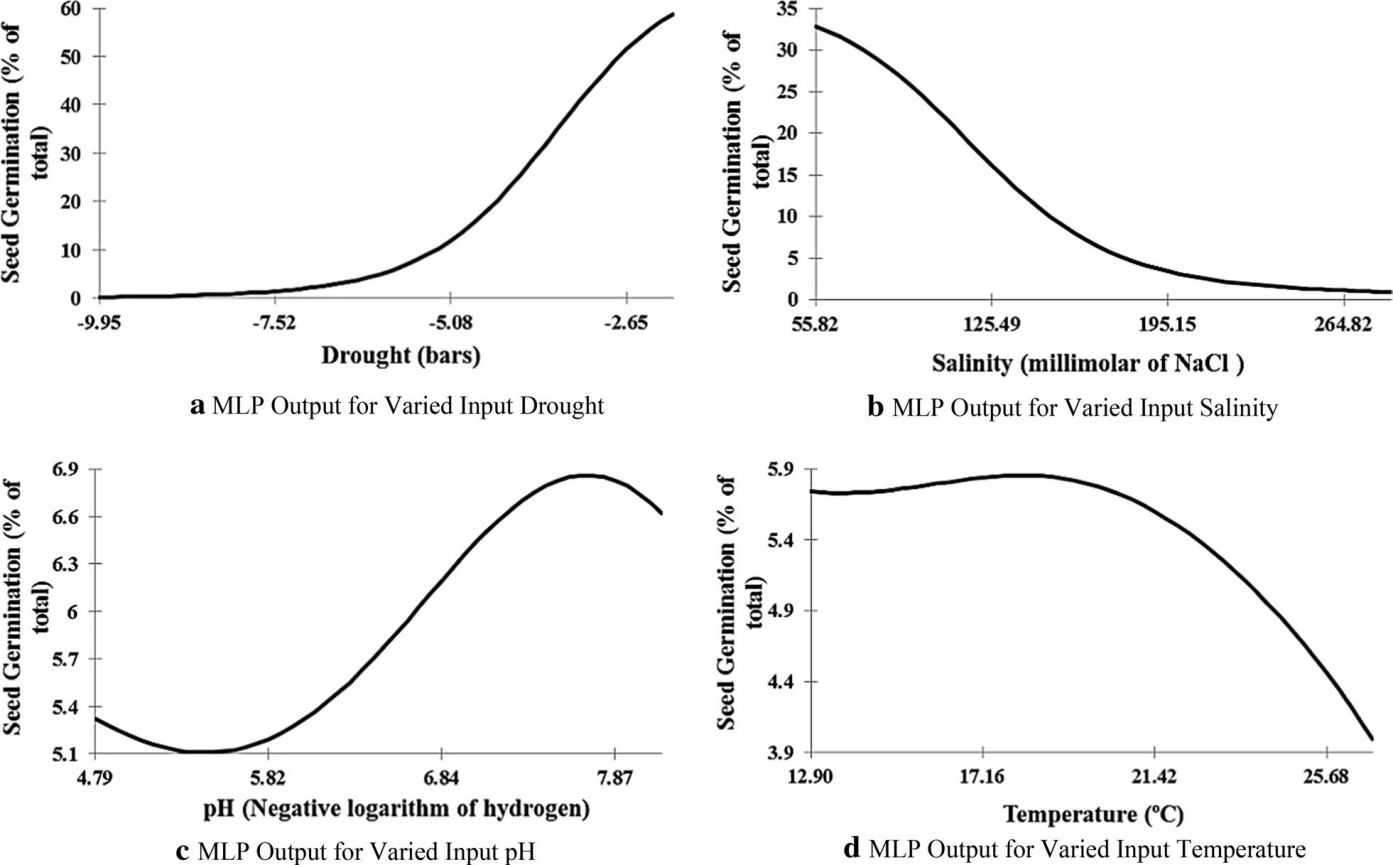
MLP Output for inputs variables

Considering trends in the Fig. 7(a), drought, is negatively correlated to seed germination, so in the ecosystems under drought stress, the percentage of *S. limbata* seed germination obviously decreases. Actually, the percentage of *S. limbata* seed germination will increase with reduction of site drought and it is happened in drought lower than −8 bars. Considering trends in the Fig. 7(b), salinity, is negatively correlated to seed germination, so in the ecosystems under salinity stress, the percentage of *S. limbata* seed germination obviously decreases. Actually, the percentage of *S. limbata* seed germination will reduce with increase of site salinity and it is happened when salinity is more than 50 millimols. The impact of pH and temperature on *S. limbata* seed germination is very limited. However, considering trend in the Fig. 7(c), pH has an optimum value for the maximum of seed germination. Indeed, the maximum of seed germination is in 7.7 pH. On the other hand, trend in the Fig. 7(d) reveals that the temperature has a maximum value for seed germination and after 18.3 °C, the percentage of seed germination decreases.

**Fig. 8:**
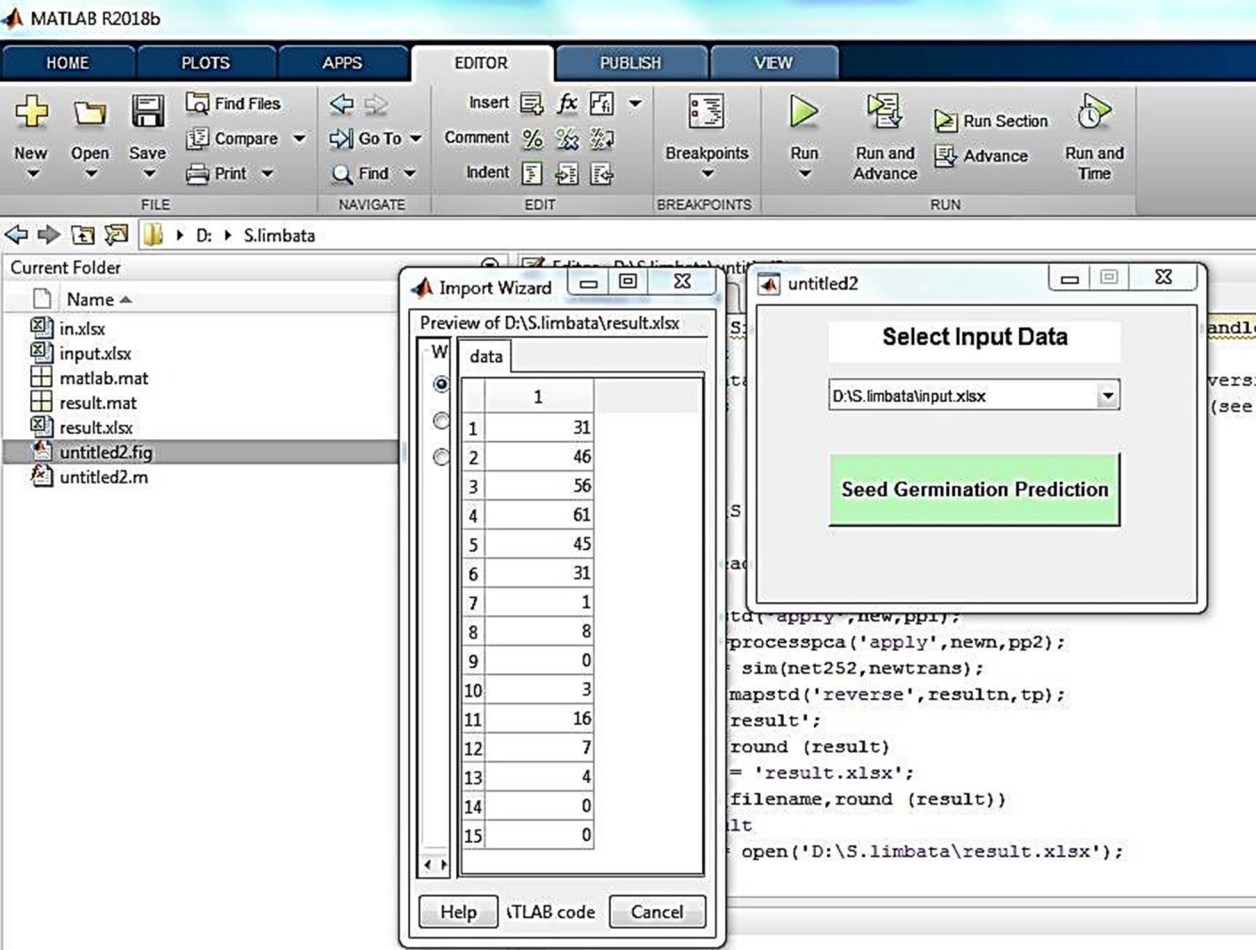
Seed germination prediction under four ecological conditions

At the end, a graphical user interface (GUI) was designed to run the MLP model on new data when the natural resources managers are planning for *S.limbata* seed planting. The manager can easily predict the percentage of seed germination under abiotic ecological stresses. GUI as an EDSS tool will be run on new data just by pushing the “Seed Germination Prediction” button in Fig. 8. As an example, Fig. 8 illustrates the results of seed germination under four ecological conditions (drought, salinity, temperature and pH).

## Discussion

The MLP model, which has been developed in this research resulted a proper description of the *S. limbata* germination dynamics in response to drought, salinity, temperature and pH. The results also provided mathematical tool to predict the percentage of seed germination under ecological abiotic stresses that has not been addressed previously. The ANN modeling approach that we applied in this research not only inherited the advantages of previous models (e.g. 24, 20, 25, 12, 56), but also included new artificial intelligence technique and some additional features as discussed below.

We compared MLR and ANN modeling techniques and we found that MLP model consistently provided better prediction of seed germination, whereas the classic models, such as MLR, was systematically biased and inaccurate [30, 35]. The results proved that MLP model (R^2^ = 0.94) is definitely more accurate than MLR (R^2^ = 0.67). The main application of MLP model is in *S. limbata* seed germination prediction based on abiotic stresses. As an environmental decision support system tool, MLP is applied for decision making of site rehabilitation in rangelands to increase the possibility of S. *limbata* seed germination. The ANN technique may be easily extended to other species, to accommodate different seed germination scheme observed in other regions or ecosystems. In a recent research, Mesgaran et al. [15] introduce a new hydrothermal time model that uses a log-logistic distribution to discover the optimum temperature changes for seed germination in respond to the water potential. They proved that optimal temperature gently decreased with reduction in water potential and log-logistic model resulted good fits to germination data. However they did not compare the results of log-logistic model with new ANN modeling techniques. We believe that MLP model can discover the relationship between ecological conditions (such as temperature and water potential in Mesgaran et al. [15]) to predict the success of seed planting. Actually, thermal-time models make an attempt to determine the base, optimum and ceiling germination in degree-days; while ecological condition tests are limited to one or two factors (refer to 29, 30, 31, 32, 28, 33]. On the other hand, ANN model in this research formulates the rate of germination under multiple ecological stress condition but there is a shortcoming in determination of base, optimum and ceiling germination degree-days. Hence, in future studies, the combination of thermal-time models and ANN models would result more accurate hybrid model which is able to determine the base, optimum and ceiling germination in multiple-stress-days. In our work, germination was described in totals, but only after 15 days (extended to 30 days after last germination). Yet, our understanding of thermal models, in which thresholds and rate of parameters are defined, tells us that beyond the optima germination becomes slower and slower. This means that under stress, 15 days of germination testing is likely insufficient to know if germination may have occurred in time, e.g. at lower temperatures. Indeed, this is the caveats associated with this particular dataset and should be considered in future work especially in suggested hybrid models of ANN and thermal models.

The results of sensitivity analysis revealed that drought and salinity are considered as the most influential factors in *S. limbata* seed germination rate; indeed, seed germination increases with the reduction of drought and salinity. Gorai et al. [57] researches also proved that the maximum of *Salvia aegyptiaca* seed germination is at 0 milli mol NaCl; and seed germination will be inhibited with the increase of salinity. Gorai et al. [57] determined 30 °C as the optimum temperature for *Salvia aegyptiaca* seed germination, but we determined 18.3 °C as the optimum temperature for *S. limbata* seed germination. Belmehdi et al. [11] determined the optimum temperature between 15 and 20 °C for *Origanum elongatu*m (one of the Lamiaceae family species) seeds germination. This result is so close to the achieved optimum temperature for *S. limbata* seeds germination at 18.3 °C in this research. On the other hand, Belmehdi et al. [11] mentioned that the optimum pH for *Origanum elongatum* seeds germination is between 6 and 7, while the optimum pH for *S. limbata* seeds germination is 7.7 in our results. Indeed, Belmehdi et al. [11] used statistical analysis (without modeling approaches) to find the optimum value of variables influencing seed germination; hence they achieved a range of values. As we modeled the seed germination by combined ecological variables, we achieved the trends of germination changes in respond to the influential variables and more crisp values were discovered.

In proposed hydrothermal time models, the researchers combine two abiotic factors to determine the effect of temperature and water potential on seed germination; but, as an advantage of ANN modeling, we combined four ecological factors to predict the percentage of seed germination. The second advantage of our research is in designed GUI tool for user application as an environmental decision support system such as the research of Bahraminejad et al. [59]. Indeed, using designed GUI, the agriculture or natural resources managers will be able to predict the percentage of *S.limbata* seeds germination under ecological constraints.

## Conclusion

In this study, a multilayer perceptron model was developed to predict the seed germination of *S. limbata* species under four ecological stresses, which helps managers to determine the success of *S. limbata* seed planting in agricultural or natural ecosystems. By using the MATLAB 2018 software, it is possible to implement the model in the regions where the ecological stresses are as the same as the tested variables in this research. Areas with high moisture content and low salinity in the soil have a high potential to seed germination of *S. limbata*. Also, the temperature of 18.3 °C and pH of 7.7 are proposed for achieving the maximum number of germinated *S. limbata* seeds.

## Methods

### Seeds collection and preparation

Mature seeds were collected in August 2019 from South Alborz protected area in Alborz province in the north of Iran (35° 44′ N to 36° 35′ N and 51° 00′ E to 51° 36′ E). Seeds were carefully gathered from mature *Salvia limbata* species. The seeds were sorted manually to avoid any ruined or injured seeds. The gathered seeds have not undergone any treatment. Indeed, the conservation was made in until their use. Indeed, germination tests were performed after 2 weeks and as Duarte et al. [28] proposed, seeds were maintained in dark and dry tubes at room temperature (25 ± 3 °C) until eventual use.

No permissions were required to collect seeds from the studied area because seeds were collected under supervision of provincial Department of Environment. There is no committee/review board or regional/national committee to approve the study; however any field study in protected areas is under supervision of provincial Department of Environment.

### Germination experiments

In order to determine the impact of different abiotic variables on *S.limbata* seed germination, a variety of parameters were studied, modeled and discussed in this research. The experiments, in different abiotic conditions, were run in sterilized petri dishes under controlled lab condition. Such as other researches (e.g. Porceddu et al. 2017), germination was defined as visible radicle emergence.

Germination percentage (%) was defined as the Eq 1 which is the ratio between germinated seeds and the total seeds.

1 $$R = \frac{n}{N} \times 100$$

In Eq 1, R is the germination percentage (%), n represents the number of germinated seeds, and N is total of seeds in the experiment [58].

In pretests, we tested seed germination in all ecological stresses one by one. Pretests revealed that the maximum time for *S.limbata* seeds germination equals 15 days. Indeed after 15 days the remained seeds begin to rot and will not germinate any more. However, in the main research tests we continued lab tests for 30 days but we did not find any new germination after day 15. As Porceddu et al. [39] proposed, a cut-test was performed to determine the viability of the ingeminated seeds and we considered soft seeds as non-viable ones.

Seeds were drowned in sodium hypochlorite solution for 1 min to be sterilized and avoid fungus attack. Before being used in the experiment, the seeds were washed with distilled water and then air-dried. The experiments were conducted in Petri dishes containing two disks of Whatman ilter paper which have been soaked with 1.5 ml of distilled water.

The *S. limbata* seeds germination was tested in different combinations of abiotic conditions containing temperature, drought, salinity, and pH. To control the effect of light, *S. limbata* seeds were incubated at light in illuminated incubator at 16/8 h photoperiod. Five different temperatures were tested which were 10, 15, 20, 25 and 30 °C to evaluate the effect of temperature on seed germination. The drought stress was tested using polyethylene glycol 6000 with seven treatments which were 0, −2, −4, −6, −8, −10 and −12 bars. Salinity stress was evaluated in eight treatments of salinity containing 0, 50, 100.150, 200, 250, 300 and 350 mM of NaCl to investigate the impact of salinity stress on germination. In order to test the effect of pH, six treatments of pH were investigated consisting of 4, 5, 6, 7, 8 and 9. *S.limbata* seeds were cultured in different combinations of abiotic conditions randomly. Indeed 228 combinations were tested to determine the percentage of germination for model development.

### Multiple regression analysis

Four independent variables, consisting of temperature, drought, salinity, and pH, were used to predict *S. limbata* seed germination. To avoid any possible bias in the selection of test set individuals, the total samples (228 treatments) were randomly divided into two subsets. Training data subset contains 80% of total samples (182 treatments), and test data subset contains 20% of total samples (46 treatments).

### Multi-layer perceptron neural network

The structure of MLP is based on the human brain by using some interconnected processing elements (PEs) or neurons. MLP learns the relations between variables (temperature, drought, salinity, and pH) and outputs (the percentage of seed germination) by the training samples. A transfer function, in the neurons, summarizes the weighted variables to predict the output while inputs of the neurons are ecosystem variables and output is the dependent target [40–42]. Indeed, the weighted input variables of each neuron are connected to other neurons variables. Using a learning algorithm, the weights of variables in hidden layers are adjusted continuously to minimize the differences between target and network output values. In this research, we applied four most popular activation functions which are hyperbolic tangent, logarithmic sigmoid, sigmoid tangent and linear transfer functions to optimize the performance of MLP. Back Propagation (BP) method is commonly used in the learning process to adjust the optimum weights and biases of neurons [34, 35, 43].

BP method aims to calculate the differences between predicted (MLP output) and target (the recorded percentage of seed germination) values for diminishing these errors. The weight of neurons (w) and input variables (x) play a main role in the observed errors, so we need to justify these parameters to reduce the differences between predicted and target values. Thus, the weights of the ith variable in jth neuron are changing continuously during training process. For the weights adjusting, the output of jth neuron on the kth hidden layer ($net_{j}^{k}$) should be calculated by Eq. **2** (refer to Demuth and Beale [44]).

2 $$\varvec{net}_{\varvec{j}}^{\varvec{k}} = \mathop \sum \limits_{{\varvec{i} = 0}}^{\varvec{n}} \varvec{w}_{{\varvec{ji}}} \varvec{x}_{{\varvec{ji}}}$$

In the next step, some transfer functions (∫), such as linear, sigmoid tangent, hyperbolic tangent, and logarithm sigmoid, are used in the structure of hidden layers and neurons output is calculated using Eq. 3. The transfer function selection is based on trial and errors to find the most accurate model and optimizing the outputs (refer to Demuth and Beale [44]).

3 $$\varvec{Y}_{{\varvec{net}}} = \smallint \varvec{net}_{\varvec{j}}$$

In this research, the weights of 228 samples will be adjusted by delta rule which has been summarized in Eq. 4. In Eq. 4, “E” is the sum of squared errors between predicted and target values, w_ji_ represent the weight of ith neuron in jth hidden layer, and γ is learning rate which is determined by a crisp value. All the weights in the hidden layers will be changed to achieve the minimum of the errors in outputs (refer to Demuth and Beale [44]).

4 $$\varvec{w}_{{\varvec{ji}}}^{\varvec{t}} = \varvec{w}_{{\varvec{ji}}}^{{\varvec{t} - 1}} + \left( { - \frac{{\partial \varvec{E}^{\varvec{t}} }}{{{}^{\gamma }\partial \varvec{w}_{{\varvec{ji}}}^{\varvec{t}} }}} \right)$$

As the errors reduce in training data set, the error of test data set may be increase and as a result, the model loses the capability of generalization which is called over training of the network. Therefore, the validation dataset are applied to control the overtraining of the MLP model. When the errors of network decrease during training process, while the errors of validation samples increase, the network training will be stopped. We divided the samples in three categories randomly which it means 60 percent of all treatments as training samples, 20 percent of all treatments as validation samples and 20 percent of all treatments as test samples. MATLAB 2018 is mathematical software which was used to design Artificial Neural Network (ANN) model and test the results.

### Modeling *S. limbata* seed germination

In order to process the data, artificial neural network intelligent in MATLAB 2018 was implemented. In this research, in order to modeling the percentage of *S. limbata* seed germination, the selected influential variables including temperature, drought, salinity, and pH were recorded as independent variables (model input), and the percentage of *S. limbata* seed germination was considered as dependent variables (model output). To train the MLP model, the samples were randomly divided into three categories of training, validation and test data sets. Training data is used to create the optimal model and is evaluated with the validation data set. Finally, the data are used to measure the generalizability and applicability of the model to the new data and determine the actual accuracy of it [34, 45, 46]. To optimize the model accuracy, the number of hidden layers, the number of and activation functions were defined by trials and errors [47–49]. Model accuracy was estimated based on the following indices: coefficient of determination (R^2^), mean absolute error (MAE), and mean square error (MSE) (Eqs. 5 to 7 [50–52]).

5 $$MSE = \frac{{\sum\limits_{i = 1}^{n} {\left( {O_{i} \,\text{ - }\,P_{i} } \right)^{2} } }}{n}$$

6 $$MAE = \frac{1}{n}\sum\nolimits_{i = 1}^{n} {\left| {\mathop O\nolimits_{i} - \mathop P\nolimits_{i} } \right|}$$

7 $$\mathop R\nolimits^{2} = \frac{{\sum\limits_{i = 1}^{n} {(\mathop O\nolimits_{i} - \mathop O\nolimits_{ave} )(\mathop P\nolimits_{i} - \mathop P\nolimits_{ave} )} }}{{\sqrt {\sum\limits_{i = 1}^{n} {(\mathop O\nolimits_{i} - \mathop O\nolimits_{ave} )\sum\nolimits_{i = 1}^{n} {(\mathop P\nolimits_{i} - \mathop P\nolimits_{ave} )} } } }}$$

In these equations: O_i_: measured data, P_i_: predicted data, O_ave_: mean measured data, P_ave_: the average of predicted data and n: number of the samples. Evaluation of the best fitting network to find the most accurate network structure was done by the above criteria which aim to maximize the coefficient of determination and minimize the mean squares of errors and mean squares of absolute error.

Each ecological variable has a particular role in the outputs value of the model [53, 54]. When we prioritize the importance of the variables, it is possible to justify the ecological variables to achieve the desired percentage of seed germination in the field. We designed a sensitivity analysis to determine the importance of each variable which influence the model output or *S.limbata* seed germination. To do this analysis, we calculated the standard deviation of each variable. Then, we changed each variable in the range of standard deviation to discover the occurring changes in model outputs. While a variable is changing in the range of standard deviation, other variables are fixed in the mean value to determine the sensitivity of model to target variable alone. The standard deviation of outputs, in respond to each variable changes, was tagged as sensitivity value for each variable (refer to Jahani et al. [41]).

Ultimately, the graphical user interface tool was developed as an environmental decision support system (EDSS) for prediction of *S. limbata* seed germination by agricultural or rangeland managers who are expecting the maximum percentage of *S. limbata* in the field.

## Supplementary information

**Additional file 1.** The provincial Department of Environment supervision letter.

## Data Availability

The datasets used and/or analysed during the current study are available from the corresponding author on reasonable request.

## Abbreviations

ANN: Artificial Neural Network
MLP: Multi-Layer Perceptron
MLR: Multiple Linear Regression
EDSS: Environmental Decision Support System
BP: Back Propagation
MSE: Mean Squared Error
RMSE: Root Mean Squared Error
MAE: Mean Absolute Error
GUI: Graphical User Interface
