# Supplementary material for: Seed germination prediction of Salvia limbata under ecological stresses in protected areas: an artificial intelligence modeling approach
Source: BMC Ecol. 2020 Aug 29;20:48. doi: 10.1186/s12898-020-00316-4 (PMC7456011; doi:10.1186/s12898-020-00316-4)
Supplement: Supplementary file 1 — Additional file 1. The provincial Department of Environment supervision letter. [file 12898_2020_316_MOESM1_ESM.pdf]

سازمان حفاظت محیط زیست

Department Of Environment

Alborz Province

Date: 2019.4.22

Number: 11/E/323

Permission approval

Considering the request of Dr. Ali Jahani, faculty member of College of Environment, for S.limbata seed collection in Alborz protected area, the provincial committee of Alborz Province Department of Environment provides permissions for seed collection under supervision of official agent who will accompany the research team.

Manager of Alborz Province Department of Environment

Fardin Hakimi

Address: Taleghani Sq. Karaj, Alborz, Iran Tel: +98 26 32523160
